# Association between Anaemia during Pregnancy and Blood Loss at and after Delivery among Women with Vaginal Births in Pemba Island, Zanzibar, Tanzania

**Published:** 2008-06

**Authors:** Justine A. Kavle, Rebecca J. Stoltzfus, Frank Witter, James M. Tielsch, Sabra S. Khalfan, Laura E. Caulfield

**Affiliations:** ^1^Department of International Health, Bloomberg School of Public Health, Johns Hopkins University, 615 North Wolfe Street, Baltimore, MD 21205, USA; ^2^Division of Nutritional Sciences, Cornell University, 120 Savage Hall, Ithaca, NY 14853; ^3^Department of Gynecology and Obstetrics, Johns Hopkins School of Medicine, 600 North Wolfe Street, Phipps 228, Baltimore, MD 21287–1228, USA; ^4^Pemba Public Health Laboratory, PO Box 122, Chake Chake, Pemba Island, Zanzibar, United Republic of Tanzania; ^5^Center for Human Nutrition, Department of International Health, Bloomberg School of Public Health, Johns Hopkins University, 615 North Wolfe Street, W2041, Baltimore, MD 21205, USA

**Keywords:** Anaemia, Blood loss, Childbirth, Community-based studies, Delivery, Postpartum haemorrhage, Pregnancy, Vaginal birth, Tanzania

## Abstract

The study sought to identify determinants of blood loss at childbirth and 24 hours postpartum. The study was nested in a community-based randomized trial of treatments for anaemia during pregnancy in Wete Town, Pemba Island, Zanzibar, United Republic of Tanzania. Status of anaemia during pregnancy, nutritional information, obstetric history, and socioeconomic status were assessed at enrollment during routine antenatal care. Pregnant women presented for spontaneous vaginal delivery, and nurse-midwives collected information on labour and delivery via partograph. Blood-stained sanitary napkins and pads from childbirth and 24 hours postpartum were quantified using the alkaline hematin method. Moderate-to-severe anaemia (Hb <90 g/L) at enrollment was strongly associated with blood loss at delivery and the immediate postpartum period, after adjusting for maternal covariates and variables of biological relevance to blood loss. Greater blood loss was associated (p<0.10) with duration of the first stage of labour, placental weight, receipt of oxytocin, preterm birth, and grand multiparity. The findings provide unique evidence of a previously-suspected link between maternal anaemia and greater blood loss at childbirth and postpartum. Further research is needed to confirm these findings on a larger sample of women to determine whether women with moderate-to-severe anaemia are more likely to experience postpartum haemorrhage and whether appropriate antenatal or peripartum care can affect the relationships described here.

## INTRODUCTION

Over half a million women die due to complications of pregnancy and childbirth each year (1). In sub-Saharan Africa where the majority of maternal deaths occur, the risk of dying in childbirth is 175 times that of developed countries (2). Postpartum haemorrhage, characterized by severe bleeding (>500 mL) after birth of the baby, is the leading cause of maternal death in African and other developing countries (3). Childbirth and the immediate postpartum period are critical time points in a woman's life, as maternal deaths primarily occur during labour, delivery, and the immediate postpartum period, with the risk of death being particularly high in the initial days following birth (4). Further, in addition to the devastating impact of maternal mortality due to postpartum haemorrhage, millions of women survive postpartum haemorrhage and continue to suffer from its debilitating consequences, including chronic illness, disability, an increased risk of death and/or poor growth and development of their children (5).

Despite the global significance of postpartum haemorrhage, little is known about factors that contribute to postpartum haemorrhage, especially in less-developed areas where 99% of maternal deaths occur. Severe anaemia may weaken uterine muscular strength or lower resistance to infectious diseases, contributing to postpartum haemorrhage and subsequent maternal mortality (6,7). However, the severity of anaemia that places a woman at a greater risk of experiencing postpartum haemorrhage or a debilitating and clinically relevant blood loss has not been investigated. Indeed, the impact of anaemia on the extent of blood lost at childbirth and postpartum is not well-understood.

Previous studies, conducted largely in non-anaemic populations, identified nulliparity, grand multiparity, obesity, birthweight of >4,000 g, lacerations/tears, episiotomy, delivery using forceps, pre-eclampsia, multiple gestation, premature birth, advanced age, short stature, induced labour, and augmented labour as risk factors for postpartum haemorrhage (8-15). Two previous studies among African populations reported no association between maternal anaemia and postpartum haemorrhage in Nigeria (8) and no difference in risk of postpartum haemorrhage between severe and non-severe anaemia among Ghanaian women (9). In contrast, cases of postpartum haemorrhage in Zimbabwe were 2.2 times more likely to have haemoglobin levels of <120 g/L than those of non-postpartum haemorrhage controls (10). However, these studies have not described the procedures of measurement of anaemia/haemoglobin and relied on visual estimation or measurement from a basin combined with visual estimation, techniques that can underestimate or imprecisely ascertain blood loss (8-10,16,17). To date, no investigations have measured blood loss at delivery and the immediate postpartum period, apart from the extremes of blood loss (postpartum haemorrhage), and examined pregnancy and childbirth determinants of blood loss.

In this study in Pemba Island, Zanzibar, United Republic of Tanzania, we examined the relationships between nutritional, obstetrical and socioeconomic factors and blood loss during childbirth and the postpartum period. Specifically, we evaluated whether maternal anaemia contributes to greater blood loss at childbirth and 24 hours postpartum.

## MATERIALS AND METHODS

Pemba Island is one of two main islands which comprises the archipelago known as Zanzibar, United Republic of Tanzania. Agriculture and fishing are the primary occupations, and the major cash crop is clove. The population is predominantly Muslim and of Bantu and Omani Arab ethnic origin. *Plasmodium falciparum* malaria and hookworm infection are endemic in the island (18,19). The typical diet consists of cassava and rice mixed with seasonally-available vegetables and fish; there is infrequent intake of meat due to poor availability and high cost (20).

This investigation was part of a larger randomi-zed trial—“Mothers and Health”—in which the effectiveness of the World Health Organization- recommended Standard Care for the prevention and treatment of severe anaemia in pregnancy was compared with an enhanced regimen. Eight antenatal clinics were randomly allocated to Standard Care and Enhanced Care by coin toss with strata defined by geographical location. Investigators, study personnel, and participants were unmasked to treatments. All women received iron-folate supplements (60 mg iron and 400 μg folate, per tablet, daily), sulphadoxine-pyrimethamine, an antimalarial drug (500 mg sulphadoxine and 25 mg pyrimethamine, per tablet, 2 doses, 4 weeks apart), and mebendazole, a deworming (Standard Care: one 500-mg dose; Enhanced Care: 100 mg, twice a day for 3 days, 2 doses, 8 weeks apart). Women in the Enhanced Care group received a daily multi-vitamin containing recommended dietary allowances of: vitamin A, 5,000 IU; vitamin E, 30 IU; vitamin C, 60 mg; vitamin D, 400 IU, thiamin, 1.5 mg, riboflavin, 1.7 mg, niacin, 20 mg; vitamin B6, 2 mg; folic acid, 400 μg; vitamin B12, 6 μg; and pantothenic acid, 10 mg.

At the baseline clinic visit, women with haemoglobin (Hb) concentration of <70 g/L received a double dosage of iron-folate tablets as per the international guidelines for iron-deficiency anaemia (21). Further details of study design, data collection, and clinical assessments are described elsewhere (22).

The sample size, based on a correlation (r=-0.20) between Hb status and blood loss, with α=0.05 and β=0.20, and a loss to follow-up of 15%, was determined to be 240 women. The first 256 eligible pregnant women attending two public antenatal clinics in Wete town from 13 April 2004 to 28 July 2004 were invited to participate in the ‘Blood Loss' sub-study at baseline antenatal visit. The inclusion criteria included: women who sought routine antenatal care at study clinics, resided within desigated study areas in Wete town, and who planned to have a spontaneous vaginal delivery with government hospital admission until 24 hours postpartum. Women presenting for cesarean delivery were excluded upon hospital admission. The final sample consisted of 158 women due to loss to follow-up as these women had either delivered prior to obtaining a blood sample, could not arrive at the hospital on time for delivery, or did not stay through the 24 hours postpartum for collection of complete data on blood loss.

All the participants were provided with a safe delivery-kit containing gloves, needles and syringes, ergometrine, oxytocin, sutures, gauze, and cotton. Additionally, each participant received a large pad to replace the plastic sheet normally used at delivery. Following delivery, sanitary pads and white underwear were distributed for collection of secreted postpartum blood.

Nurses at the antenatal clinic and study staff collected information on nutritional (weight, height) and socioeconomic variables (e.g. household possessions, educational level) at baseline. Parity was defined as the number of previous births. For analyses, parity was categorized into four groups: nulliparous, 0 previous birth (reference category), primiparous (1 previous birth), multiparous (2–5 previous births), and grand multiparous (≥5 previous births).

The study nurses collected blood samples (5 mL) by venipuncture in heparinized Vacutainer tubes at baseline and 34–36 weeks gestation. Zinc protoporphyrin was measured by haematoflourometre. Drops of whole blood were dispensed to make thick malaria blood films at baseline and 36 weeks gestation and to determine haemoglobin concentrations at baseline, 32 weeks, and 36 weeks gestation. Women with severe anaemia (Hb <70 g/L), were asked to return to the clinic one week and four weeks post-baseline for clinical evaluation of improvement in haemoglobin concentrations and hospital referral, if needed. Malaria smears were fixed in alcohol and stained with Giemsa, and the number of malaria parasites was counted against leucocytes. Faecal samples were examined for helminth infection using the Kato Katz method (23).

For collection of secreted blood at delivery and postpartum, a hospital setting was chosen for ease in collection and storage of pad samples. The timeframe of data collection (24 hours following the time of delivery) followed the American College of Obstetrics and Gynecology and World Health Organization's definition of early postpartum haemorrhage (3,24).

A large thin absorbent white pad was placed underneath the woman following pelvic examination to confirm full dilation. Ergometrine maleate (0.5 mg/mL) was routinely given as part of active management of the third stage of labour, after the birth of the baby. As part of hospital protocol, oxytocin (1, 2, 3, or 5 IU) was administered to either augment labour or reduce excessive bleeding. Gauze, cotton, and white gloves were used for mopping up excessive blood loss at delivery. Following placental delivery, the nurses held the placenta to collect maternal blood onto the delivery pad and weighed the placenta using a scale. Secreted blood at childbirth was collected from full dilation until delivery of the placenta.

Postpartum collection of secreted blood commenced after the third stage of labour and ended at 24 hours post-delivery. Immediately following the removal of delivery pad, the nurses and orderlies initially assisted women for the collection of postpartum blood. Orderlies provided women with sanitary pads and white cotton underwear for the remaining 24 hours, which were collected in Stomacher bags and placed in a cooler after each collection. All delivery and postpartum samples were transferred to the hospital laboratory. Blood loss was measured using the alkaline hematin technique (22,25).

Two litre of a 5% sodium hydroxide solution (J.T. Baker, Phillipsburg, NJ, USA) was added to each pad sample. Samples were then placed in the Stomacher Blender (Seward Company, Norfolk, UK) for 15 minutes. The automated paddles of the blender facilitated breakdown of pad material and conversion of haemoglobin to an alkaline hematin solution, which was decanted into a glass beaker. The weights of all materials added to Stomacher bags were subtracted from calculations of total blood loss. Further details on quantification methods of blood loss are described elsewhere (22,25).

### Ethics

The human subject review committees at the Johns Hopkins University Bloomberg School of Public Health, Baltimore, Maryland, USA, Cornell University, Ithaca, New York, USA, and the Zanzibar Research Council, United Republic of Tanzania, independently approved this project.

### Statistics

To evaluate anaemia during pregnancy, we used haemoglobin cut-offs: 110 g/L, 105 g/L, and 110 g/L for baseline, 32 weeks, and 36 weeks gestation respectively. ‘Moderate'-to-‘severe' anaemia was defined as Hb <90 g/L. Additionally, we evaluated zinc protoporphyrin—an indicator of iron deficiency—using an international cut-off for iron-deficient erythropoiesis measured by erythrocyte protoporphyrin as >70 μmol/mol heme (26). Erythrocyte protoporphyrin usually exists as zinc protoporphyrin, the major molecular form of erythrocyte protoporphyrin (27).

We computed compliance with iron-folate and multivitamin supplementation, mebendazole (one dose or two doses), and Fansidar (baseline and/or four weeks after baseline). Home monitors counted the ‘number of pills remaining' for iron or iron plus multivitamin pills per bi-weekly period until childbirth. We calculated ‘percent compliance' for iron and multivitamins (restricting computations from enrollment until childbirth) using the following formula:

$\frac{\begin{array}{l} \text{Total number of pills given until childbirth}-\\ \text{total number of pills remaining} \end{array}}{\begin{array}{l} \text{Total number of pills given to women from}\\ \text{enrollment until childbirth} \end{array}}$

In our analysis, we explored nutritional, obstetrical and socioeconomic factors as determinants of blood loss. We examined the relationships between total blood loss (millilitre of blood loss at childbirth and postpartum) and factors previously reported to be associated with postpartum haemorrhage. We used simple linear regression, Wilcoxon rank sum, and Kruskal-Wallis tests for bivariate analyses. Specifically, to determine the relationship among maternal anaemia, iron deficiency, and total blood loss, we considered the relationship between total blood loss and measures of iron status, including anaemia and found a linear specification to be appropriate. Then, we assessed the relationship between categories of severity of anaemia and zinc protoporphyrin levels and total blood loss. The relationships with changes in haemoglobin values from recruitment to 36 weeks gestation were also evaluated.

For our multivariate analyses, each variable was evaluated and retained in the model based on the strength of association with blood loss, as ascertained by p value. Variables identified in previous investigations and/or had biological relevance to blood loss at childbirth were retained in our model. For example, the presence of an episiotomy or vaginal tear was retained in our final model due to prior consistent associations of blood loss with perineal lacerations or operative episiotomy. For variables with more than two categories, a series of dummy variables were created (e.g. parity). Variables associated with total blood loss (mL), at p<0.10, were included in our final model. A higher alpha value allowed for adequate examination of all related determinants, which would otherwise be restricted at lower values (e.g. p<0.05).

## RESULTS

The median age of participants was 25.5 years, and the median gestational duration was 29.0 weeks (Table 1). The majority (63.2%) of women were multiparous, with 37% of women reporting 2–5 previous pregnancies and 26% of women reporting ≥5 previous pregnancies. Women had relatively low rates of intestinal helminth infection, most likely due to schistosomiasis and filariasis-deworming campaigns (25–26 April 2004 and 18–19 October 2003 and 2004 respectively) that distributed albendazole and ivermectin to all non-pregnant Zanzibari adults.

**Table 1:** Selected characteristics of 158 Zanzibari women at enrollment

Baseline characteristics	50^th^ (5^th^, 95^th^) or %
Age (years)	25.5 (18.0, 38.0)
Gestational age (weeks)	29.0 (20.0, 37.0)
Trimester	
Second	18.4
Third	81.6
Maternal weight (kg)	59.6 (45.2, 83.2)
Maternal height (cm)	155.6 (147.6,167.0)
Maternal BMI (kg/m^2^)	24.4 (19.1, 34.1)
Haemoglobin (g/L)	105 (79, 140)
Haemoglobin category (g/L)	
No anaemia ≥110	38.6
Mild anaemia 90–109	45.6
Moderate—severe anaemia <90	15.8
Parity (%)	
0	21.5
1	15.2
2–5	37.3
≥5	25.9
Malarial infection (%)	3.8
Intestinal helminth infection* (%)	
*Trichuris*	26.5
Hookworm	13.9
*Ascaris*	2.0
Employment (%)	
No	80.4
Yes	19.6
Education (%)	
No school	3.2
Quranic school only	9.5
Completed some primary education	17.1
Completed primary education	17.1
Secondary, form 1–4	52.5
Secondary, form 5–6	0.6
Possession of household items (%)	
Radio	79.7
Bicycle	57.0

*7 missing, n=151 for helminth variables; BMI=Body mass index

All were singleton spontaneous deliveries, with the exception of three women who delivered twins (Table 2). Most (95.6%) women received ergometrine, and about a half received oxytocin in the third stage of labour. Our data revealed a low rate of tears and episiotomy. The average birthweight was 3,410±434 g.

**Table 2:** Selected labour and delivery characteristics of 158 Zanzibari women

Characteristics at childbirth	50^th^ (5^th^, 95^th^) percentile or %
Received any oxytocin (%)	44.9
Received any ergometrine (%)	95.6
Received both ergometrine and oxytocin (%)	40.5
Tear (%)	7.6
Episiotomy (%)	16.5
Duration (hours) of labour	
Nulliparous	11.0 (5.8, 22.5)
Primiparous	8.0 (5.8, 15.9)
Multiparous	6.8 (3.2, 15.4)
Grand multiparous	6.5, (4.4, 13.8)
Gestational age (weeks)	39.1 (36.4, 43.5)
Birthweight (g)*	3,410±434

*Mean±SD, calculated for singleton births only (n=155); SD=Standard deviation

Our findings revealed that 75% of women with Hb <110 g/L had the zinc protoporphyrin values of >70 μmol/mol, indicative of iron-deficient erythropoiesis and presumably iron deficiency. Moreover, all women with Hb <90 g/L had the zinc protoporphyrin values of >70 μmol/mol. The intensity of hookworm infection was negatively associated with Hb (p<0.01).

Women were compliant with the iron-folate and multivitamin supplements, on 95.4% and 97.9% of days respectively. Less than half of the participants were given Fansidar (39.0% at baseline and 42.0% at second dosing, four weeks post-baseline) due to self-reported sulpha drug allergies, gestational age >34 weeks, or early pregnancy (no evidence of foetal movement or foetal heartbeat or fundal height less than 16 weeks gestation). Approximately 92.4% of the women took one dose of mebendazole during the course of the study. Of the Enhanced Care enrollees, 96.1% received a second dose of mebendazole, eight weeks post-baseline.

In bivariate analyses, increased blood loss at childbirth and postpartum was strongly associated with the severity of maternal anaemia at enrollment (p=0.02) and at 32 weeks gestation (p=0.03) (Table 3). The change in haemoglobin concentration from recruitment to 36 weeks gestation was not associated with total blood loss.

**Table 3:** Associations of anaemia during pregnancy and iron deficiency with total blood loss in 158 Zanzibari women

Anaemia during pregnancy and iron deficiency as determinants of blood loss	p value	Median volume (mL) of blood lost at childbirth and postpartum (25^th^, 75^th^ percentile)
Baseline haemoglobin*, n=158 ≥110 g/L, n=61 90–109 g/L, n=72 <90 g/L, n=25	0.02	218 (155, 303) 247 (168, 300) 301 (222, 441)
Change in haemoglobin from recruitment to 36 weeks gestation* (g/L) by quartile, n=137 −50 to −2.0, n=36 −2.0 to 5.0, n=38 5.0 to 16, n=35 16 to 60, n=38	0.58	216 (152, 312) 230 (161, 296) 272 (187, 412) 267 (204, 327)
Baseline ZPP^†^ (μmol/mol heme), n=158 <70, n=44 >70, n=114	0.30	224 (162, 299) 262 (176, 329)
32 week haemoglobin*, n=85 ≥105 g/L, n=44 90–104 g/L, n=35 <90 g/L, n=6	0.03	194 (134, 280) 252 (211, 325) 314 (135, 373)
36 week hemoglobin*, n=137 ≥110 g/L, n=74 90–109 g/L, n=56 <90 g/L, n=7	0.71	249 (170, 321) 238 (163, 317) 311 (135, 441)
Treatment group^‡^ n=159 Standard Care, n=77 Enhanced Care, n=81	0.37	256 (187, 325) 248 (150, 321)

*Kruskal-Wallis non-parametric test

^†^Simple linear regression

^‡^Wilcoxon Rank sum test; ZPP=Zinc protoporphyrin

Increased total blood loss was also associated with the greater duration of the first stage of labour (p=0.02), receipt of oxytocin during the third stage of labour (p=0.06), and first—degree tear at childbirth (p=0.08). Women with tears (p=0.08) experienced greater losses than women without any trauma at delivery. Blood loss was lower among those delivering preterm (<37 weeks gestation) (p=0.01). These data are not presented in tabular form.

In multivariate analysis, women with moderate-to-severe anaemia at enrollment had a significantly greater total blood loss (91 mL) average compared to non-anaemic women (p<0.01) (Table 4). Blood loss was slightly elevated in mild anaemic women compared to non-anaemic women; however, this association was not statistically significant. Greater blood loss was associated with an increased duration of the first stage of labour (p=0.01), greater placental weight (p=0.03), and receipt of oxytocin with ergometrine (p=0.05) (Table 4). The latter association could be due to the use of oxytocin to reduce bleeding in diagnosed cases of postpartum haemorrhage. Also, a lower dosage of oxytocin was administered to women, as 73% received 1 IU instead of the 10 IU normally given. Suboptimal dosing was unlikely to reduce blood loss effectively.

**Table 4:** Determinants for total blood loss (mL) at childbirth and 24 hours postpartum estimated by multiple linear regression*, n=153, R^2^=0.27

Independent variable	Blood loss (mL) (SE)	p value
Haemoglobin at recruitment <90 g/L*	90.6 (28.4)	<0.01
90–110 g/L >110 g/L (reference)	11.5 (20.5) -	0.56
Placental weight (per 100 g)	11.0 (5.0)	0.03
First stage of labour (hours)	7.1 (2.8)	0.01
Oxytocin received	39.6 (19.7)	0.05
Preterm birth <37 weeks	-52.5 (29.9)	0.08
Parity 0 (reference)	-	
1	34.2 (34.2)	0.32
2–5	30.3 (33.0)	0.36
≥5	59.6 (35.2)	0.09
Tear	25.0 (37.6)	0.51
Episiotomy	18.8 (34.0)	0.58
Standard Care Enhanced Care (reference)	1.9 (19.3) -	0.92

*Adjusted for socioeconomic factors (presence of household latrine, ownership of motorcycle); SE=Standard error

Preterm birth was associated with decreased blood loss (p=0.08) compared to deliveries at term. Primiparous and multiparous women had blood losses of similar magnitude, yet grand multiparous women experienced the greatest blood loss (p=0.09) compared to nulliparous women.

Treatment assignment was not associated with blood loss, and there was no evidence for interaction between treatment assignment and baseline haemoglobin on the level of blood loss.

## DISCUSSION

Our most salient finding in this study is a strong association between moderate-to-severe anaemia at 28 weeks gestation (on average) and greater severity of blood loss at delivery and postpartum. This association is noteworthy because it persisted after adjustment for confounding factors, including labour and delivery variables, placental size, obstetric history, socioeconomic status, and treatments of anaemia. The association persisted at 32 weeks gestation, but not at 36 weeks gestation.

Our bivariate data suggest increased total blood losses with elevated zinc protoporphyrin, particularly at values of 70 μmol/mol heme or greater. Zinc protoporphyrin is a measure of iron deficiency, and most women with Hb <90 g/L had elevated zinc protoporphyrin as did the majority of women with Hb <110 g/L. It is true, however, that the zinc protoporphyrin values are also elevated with infection, malaria, and haemoglobinopathies, e.g. sickle-cell anaemia (27-30), and elevated zinc protoporphyrin and, thus, does not confirm that our association is restricted to those with iron-deficiency anaemia.

We found no effect of treatment of anaemia on total blood loss, as both the treatment groups showed similar blood losses and haemoglobin concentrations. Further, we found no significant difference by treatment group in the change in haemoglobin from enrollment (28 weeks gestation on average) to 36 weeks gestation. All participants received the same dose of iron and folic acid; therefore, any additional benefit conferred by the treatment group would be due to the multivitamins received by women in the Enhanced Care group. The fact that the association was the strongest among those in mid-pregnancy might suggest that our results are due to plasma volume expansion, if women with greater haemodilution (and lower haemoglobin concentrations) during mid-pregnancy experience more blood loss. A greater understanding of the influence of biological pathways on blood loss at childbirth and postpartum will be required to refine our interpretations.

Several biological mechanisms are thought to play a role in postpartum haemorrhage. Higher blood loss may be attributed to impaired uterine muscle strength for labour when it is prolonged, or decreased resistance to infection, as infection is suggested to contribute to uterine dysfunction or inertia (6,7). In our study population, we had a few cases of prolonged labour and no cases of uterine infection or fever. Decreased uterine blood flow or low uterine muscle strength may contribute to inefficient uterine contractions and contribute to blood loss, potentially mediated by low body iron stores (serum ferritin <100 μg/L) and, therefore, iron-deficiency anaemia (31,32). Our data are consistent with this latter hypothesis. Severe anaemia is hypothesized to impair tolerance of postpartum haemorrhage and contribute to maternal death, possibly due to the failure of women to endure such excessive blood losses and late arrival at admission (33,34). In our study, the associations between blood loss and maternal anaemia emanate from women with timely hospital admission, normal labour and delivery, and a few occurrences of severe bleeding. These data suggest the influence of maternal anaemia on blood loss at childbirth and postpartum is more pervasive, affecting blood loss across a normal range of losses. Thus, our findings are likely to be underestimates of the effect of anaemia on blood loss in rural Africa, given the data are collected on women residing in town areas with hospital-based births, rather than home-based deliveries where access to healthcare may be limited, and chronic, untreated anaemia is likely to occur.

The present study had several limitations. The relationship between anaemia and blood loss at childbirth and postpartum was examined in women whose blood loss was reduced by oxytocic drugs, limiting the generalizability of our findings. We did not determine other measures of iron status or markers of infection to conclusively determine iron deficiency as the primary cause of anaemia. We also had a relatively large loss to follow-up as many women did not get to the hospital prior to delivery. We chose a hospital-based sample due to ease in material collection, processing, and cultural sensitivities to home-based postpartum blood collection. However, since the majority of African women deliver at home due to personal preference, failure to seek care, limited access and/or receipt of timely obstetric care, our findings require confirmation in home-births characterized by little to no management of labour and delivery. Minimal medical care may result in an elevated risk for greater blood loss and postpartum haemorrhage (35).

However, given the lack of reliable, high-quality data on blood loss at childbirth in sub-Saharan Africa where the problem of maternal mortality is most acute, these data are an important step in elucidating the impact of anaemia on the health and survival of women. Our findings provide unique evidence of a previously-suspected link between maternal anaemia and greater blood loss at childbirth and postpartum. Further research is needed to confirm these findings on a larger sample of women to determine whether women with moderate-to-severe anaemia are more likely to experience postpartum haemorrhage and whether appropriate antenatal or peripartum care can affect the relationships described here.
